# Supplementary material for: Friend or Foe? Exploring the Role of Cytomegalovirus (HCMV) Infection in Head and Neck Tumors
Source: Biomedicines. 2024 Apr 15;12(4):872. doi: 10.3390/biomedicines12040872 (PMC11048144; doi:10.3390/biomedicines12040872)
Supplement: Supplementary file 1 [file biomedicines-12-00872-s001.zip › biomedicines-2926144-supplementary.pdf]

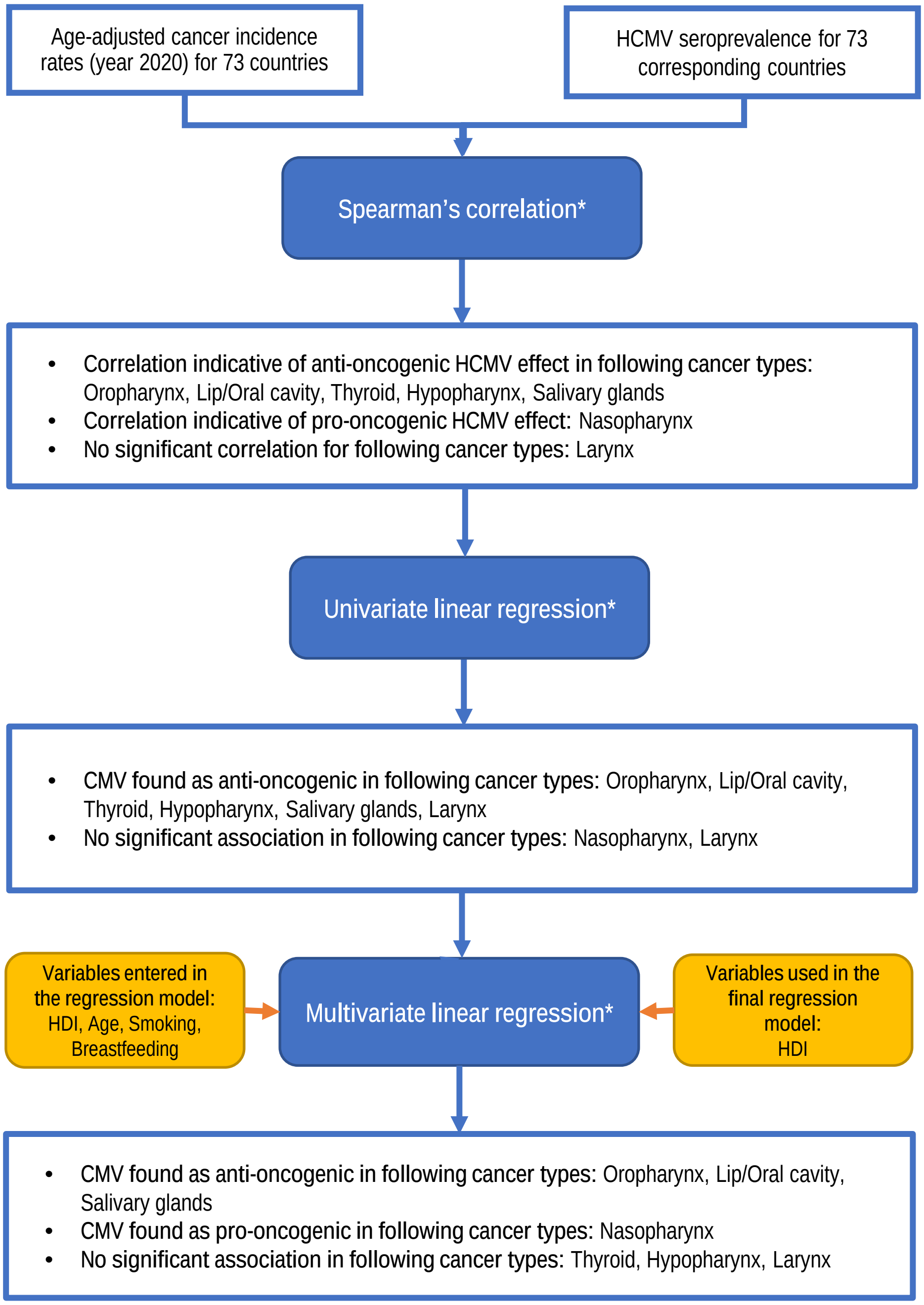

Supplementary Figure S1. The flowchart represents the steps in statistical analysis used to investigate the association between tumor incidences and HCMV seroprevalences in specific countries. The first approach used Spearman's correlation to ascertain the link between specific tumors and respective HCMV pervasiveness. Next, univariate regression analysis was used to inspect the relationship between HCMV and inspected cancer types incidence. Lastly, multivariate regression accounted for specific HCMV-oriented confounders that could compromise the veracity of correlated results. Detailed statistical results are complemented in Table 2.
